# Systematic evaluation of laryngeal impairment in Sjögren’s syndrome

**DOI:** 10.1007/s00405-021-06746-0

**Published:** 2021-03-25

**Authors:** S. Graf, L. Kirschstein, A. Knopf, N. Mansour, O. Jeleff-Wölfler, A. M. S. Buchberger, B. Hofauer

**Affiliations:** 1 Otorhinolaryngology/Phoniatrics, Klinikum rechts der Isar, Technical University Munich, Ismaningerstr. 22, 81675 Munich, Germany; 2 Otorhinolaryngology/Head and Neck Surgery, University Medical Center Freiburg, University of Freiburg, Freiburg im Breisgau, Germany

**Keywords:** Sjögren’s syndrome, Dysphagia, Dysphonia, Quality of life, Voice examination

## Abstract

**Introduction:**

Sjögren’s syndrome (SjS) causes malfunction of the salivary and lacrimal glands. Consequently, patients suffer from xerostomia and keratoconjunctivitis sicca. This can further affect the voice and swallowing function resulting in an impaired quality of life. Aim of this study is the systematic evaluation of the impact on voice and swallowing-related quality of life in patients with SjS.

**Material and methods:**

SjS patients were classified according to the American–European Consensus Group (AECG) criteria; antibodies to Ro (SS-A) or La (SS-B) antigens were detected, ESSPRI was completed. We used the following quality of life questionnaires: EORTC QLQ H&N 35, Anderson Dysphagia Inventory (ADI) and Voice Handicap Index (VHI). Patients additionally received a detailed phoniatric examination (auditory perception, videostroboscopy, acoustic analysis, Dysphonia Severity Index (DSI), aerodynamics measurements).

**Results:**

Almost all the 54 patients (96.3%) had a limited quality of life due to their swallowing problems and 48% due to their voice problems. Both values correlated significantly with the degree of xerostomia. In the phoniatric examination, 77.8% had an increased DSI and two-thirds had abnormalities in videostroboscopy.

**Conclusions:**

A reasonable impairment of quality of life in patients with SjS due to the limitations in voice and swallowing function was observed. As SjS does not limitate life expectancy, preservation of quality of life is important. Detection of voice and swallowing problems as potential reasons for quality of life impairment should be detected and, if diagnosed, treated accordingly.

## Introduction

Sjögren’s syndrome (SjS) is a systemic, chronic autoimmune disorder that affects primarily the salivary and lacrimal glands. It is known that white patients are more frequently affected by the glandular domain compared to people of African American origin, Asian and Hispanic patients [4, 5].

The cardinal glandular symptoms are xerostomia, keratoconjunctivitis sicca and parotidomegaly [6, 7]. Diagnosis is nowadays usually based on the American College of Rheumatology/European League against Rheumatism classification criteria for primary SjS [8]. SjS induces disorders of the functions of mouth, throat and larynx. This causes disorders in swallowing and in voice [9].

Dryness in SjS patients leads to dysphagia, with the major impact on oral and pharyngeal transport. Often patients have to drink at mealtime to remove leftovers from the mouth. Frequently, food leftovers (residuals) are observed in the pharynx. These have to be cleaned by the patients by clearing the throat with great effort, repeated swallowing and/or drinking, which causes prolonged meal times. This can have serious consequences, such as malnutrition/–nourishment and dehydration [10], swallowing disorders lead to a decreased quality of life, regardless of their etiology. [11].

SjS can also cause voice problems [12]. Voice production is a complex phenomenon that involves not only the larynx but the entire vocal tract. The vocal tract is formed by the spaces of the throat, the nasal and oral cavity. This resonance area modulates the voice waves of the larynx and produces speech sounds. Voice disorders result from dysfunctions of these structures [13]. Changes in the vibrational properties of these structures, such as those caused by SjS, lead to voice disorders [14, 15]. Voice is an important form of expression in our communication society. Therefore, diagnosis and therapy of voice disorders are relevant.

SjS is a chronic disease. A causal therapy is quite problematic. It is therefore even more important that patients maintain a good quality of life [16]. Both, swallowing and voice problems, are associated with a reduction of the quality of life [17].

The aim of the study is to evaluate both the swallow- and voice-related quality of life in Sjögren's patients. Furthermore, we performed a detailed multidimensional voice examination. In contrast to the previous studies, the relationship between the voice and swallowing disorders and the discomfort caused by the SjS is further analyzed.

## Materials and methods

### Study population

Patients who were referred with xerostomia and keratoconjunctivitis sicca to the Department of Otorhinolaryngology of the Technical University Munich during February 2018 to December 2018 and were classified as primary SjS were included in this study. The patients were either presented to our outpatient clinic due to symptoms in the ENT area (dry mouth, burning of the tongue, swelling of the salivary glands) or were consulted by colleagues from the rheumatology department for assessment. Classification has been done according to the American–European Consensus Group (AECG) criteria [18]. Patients with past head and neck radiation, Hepatitis C infection, AIDS, pre-existing lymphoma, sarcoidosis, graft versus host disease or the recent use of anticholinergic drugs had been excluded.

Subjective complaints (xerostomia, keratoconjunctivitis sicca, parotidomegaly) were evaluated with visual analogue scales (VAS). Salivary gland involvement was further assessed with the measurement of unstimulated whole salivary flow (UWSF). Antibodies to Ro (SS-A) or La (SS-B) antigens were detected. If necessary, minor salivary gland biopsy has been performed through a vertical incision of normal appearing mucosa of the lower lip. A minimum of five minor salivary glands had to be obtained and histopathological examination has been done according to the score proposed by Chisholm and Mason [19].

### Quality of life questionnaires

The quality of life is associated with different impaired laryngeal functions. For this purpose, we used standardized and validated quality of life questionnaires.

#### ESSPRI

The EULAR Sjogren’s Syndrome Patient Reported Index (ESSPRI) was completed by the patients and it contains three items to be given an activity level score between 0 and 10: pain, fatigue and dryness, the final ESSPRI score is the mean of all three scores and therefore also between 0 and 10 [20].

#### EORTC QLQ H&N 35

The QLQ H&N35 incorporates nine multi-item scales: five functional scales (physical, role, cognitive, emotional, and social); three symptom scales (fatigue, pain, and nausea and vomiting); and a global health and quality-of-life scale. Several single-item symptom measures are also included [21].

#### The Anderson Dysphagia Inventory (ADI)

The German version of the ADI consists of 20 questions from the global, emotional, functional and physical domain. Each question can be scored 1–5 points. A value of less than 55 is considered “highly noticeable”, 55–70 “rather noticeable” and greater than 70 “not noticeable” [22].

#### The Voice Handicap Index (VHI)

The validated German version of the VHI measures voice-related impairment of the quality of life in the functional, physical and emotional dimension.

[23]. VHI values of 0–11 are classified as grade 0 suffering (almost certainly not noticeable), while values of 12–28 reflect grade 1 suffering (more likely unnoticeable than conspicuous); values of 29–56 reflect grade 2 suffering (more probably noticeable than not), and values of 57–120 suggest a classification of certainly noticeable and are graded as grade 3 suffering [24].

### Voice assessment

Patients were offered an additional detailed examination of the voice functions on a voluntary basis as an extension to the aforementioned examinations. The laryngeal assessment was carried out according to the protocol of the European Laryngological Society [3]. The tests were carried out by experienced speech and language therapists and phoniatricians. The auditory perception was performed by the parameters grade (overall degree of voice deviance from normal), roughness (irregular fluctuation of the fundamental frequency), breathiness (turbulent noise produced by air leakage), asthenia (overall voice weakness), and strain (impression of tenseness or excess effort when speaking). Each parameter was scored on a scale of 0–3 (0 = normal; 1 = slight disturbance; 2 = moderate disturbance; 3 = severe disturbance). The instrumental examination was visualized by videostroboscopy whereby the parameters glottis closure, amplitude, regularity, mucous membrane movements and symmetry were measured.

In the acoustic analysis we determined jitter, voice range profile (phonetogram) and Dysphonia Severity Index (DSI). Jitter is defined as the parameter of frequency variation from cycle to cycle. We have slight modulations in pitch when we speak in our natural language, otherwise we would sound monotone. In the case of voice disorders these are increased. Vocal range profile (VRP) is a representation of a person’s minimum and maximum intensity levels across his/her vocal range and phonetograms are the graphic illustrations of these. Normally the range of the voice is at least two octaves.[25]. DSI is calculated using a weighted combination of four vocal parameters to provide an objective and quantitative measure of voice quality, including jitter percentage, the highest frequency and the lowest intensity of a voice range profile, and maximum phonation time (MPT) [26]. MPT is the longest period during which a patient can sustain phonation of a vowel sound, typically/a/[27]. Aerodynamics were quantified with the phonation quotient. The ratio of vital capacity (VC)-to-MPT (VC/MPT) will provide the measurement of phonation quotient (PQ) in milliliters per second (mL/s) [28].

### Statistical analysis

Version 25.0 of the Statistical Package for the Social Sciences software (SPSS, Chicago, IL, USA) was used. Descriptive statistics were calculated for demographic variables. Mann–Whitney *U* test was used to compare mean values. Data are given as mean ± standard deviation. Pearson correlation coefficient R was used for the analysis of correlations (0.80–1.00 = very strong correlation, 0.60–0.79 = strong correlation, 0.40–0.59 = moderate correlation, 0.20–0.39 = weak correlation, 0.00–0.19 = very weak correlation). *p* values of ≤ 0.05 were considered statistically significant.

The local ethics committee (Faculty of Medicine, Technical University Munich) approved this study. Informed consent was obtained from every patient.

## Results

### Patients’ characteristics

A total of 54 patients were examined (Table 1). Median age was 58.3 years (21–87). The average duration of the disease was 7.2 months with a maximum of 42 months. There were 5 men and 49 women included. About 37% of the patients were positive in the tests for antibodies to Ro (SS-A) and nearly 26% for antibodies to La (SS-B). 100% of patients received a biopsy, of which 92% were classified as positive (Focus score of 3 and 4 according to Chisholm and Mason).

**Table 1:** Sjögren’s syndrome-related parameters of subjects *n* = 54

Age (years)	58 [21–87]
Gender, male/female	5/49
Disease duration (months)	7.2 [0–42]
UWSF (mL/5 min)	0.67 [0-2, 034]
Antibodies to Ro (SS-A)	pos. 37.04%
Antibodies to La (SS-B)	pos. 25.93%
Biopsy	pos. 91.67%
Xerostomia	31.74 [1–60]
ESSPRI (total)	5.06 [1-9, 33]
ESSPRI (dryness)	5.73 [0–10]
ESSPRI (fatigue)	5.23 [0–10]

All 54 patients completed the ESSPRI questionnaire with the subcategories pain, fatigue, and dryness. The highest values could be measured for dryness.

### Quality of life questionnaires

The quality of life assessment studies showed, that many patients are restricted in their quality of life.

All patients had an increased score of the EORTC QLQ H&N 35 of at least 37–77 points, with a mean average score of 56 points. Almost all patients (96.3%) were moderately to severely impaired by their swallowing problems. The limitations also applied to their voice problems, but to a lesser extent (48%) (Table 2).

**Table 2:** Results of voice handicap index and anderson dysphagia inventory (*n* = 54)

	None	Low-grade/not noticeable	Moderate/rather noticable	High-grade/highly noticeable
Voice handicap index	28 (52%)	16 (29.63%)	7 (12.96%)	3 (5.55%)
Anderson dysphagia inventory	0 (0%)	2 (3.7%)	15 (27.78%)	37 (68.52%)

The correlation between the parameters was investigated. We found that there was a statistically significant correlation between ESSPRI, xerostomia and the impairment of quality of life in the areas of voice and swallowing.

We found a direct correlation between the grade of xerostomia and the limitation of the quality of life for all areas studied. The same was true for the duration of disease and the decrease in salivary flow (*R* =  − 0.306, *p* = 0.025).

The swallowing and the voice-related quality of life indicated a strong correlation between aspects of SjS disease and the different domains of quality of life. The ADI correlated strongly with xerostomia (*R* =  − 0.782, *p* < 0.001), ESSPRI (*R* =  − 0.501, *p* < 0.001) furthermore with the VHI (*R* =  − 0.716, *p* < 0.001) and EORTC QLQ H&N 35 (*R* =  − 0.637, *p* < 0.001). The VHI correlated with xerostomia (*R* =  − 0.510, *p* < 0.001) and ESSPRI (*R* = 0.458, *p* < 0.001), not with salivary flow, but strongly with the ADI (*R* =  − 0.716, *p* < 0.001) and EORTC QLQ H&N 35 (*R* =  − 0.618, *p* < 0.001).

### Voice parameters

In 18 patients a detailed voice examination was performed. First, the aerodynamic was evaluated. The majority of the patients had a too low maximal phonation time (MPT) (67%) with a mean value of 13.86 and a standard deviation of 6.19. The results of the vital capacity (VC) showed 39% to be too low and 61% to be higher than the normal value. Accordingly, the phonation quotient (PQ = VC/MPT) was normal in 50% of the patients (Table 3).

**Table 3:** Aerodynamic parameters (*n* = 18)

	Below normal	Above normal	Normal
Vital capacity	5 (38.5%)	8 (61.5%)	–
Phonation quotient	1 (7.1%)	6 (42.5%)	7 (50%)

Further, an acoustic analysis was conducted. In the phonetogram, most patients were able to achieve a normal pitch range (56%) and dynamic range (72%). Jitter resulted in abnormal findings in half of the patients (9/50%) (Table 4).

**Table 4:** Acoustic analysis (*n* = 18)

Voice range profile	Below normal	Above normal	Normal
Frequency range	7 (38.9%)	1 (5.6%)	10 (55.6%)
Intensity range	8 (47.1%)	6 (35.3%)	3 (17.6%)
Maximum frequency	7 (38.9%)	1 (5.6%)	10 (55.6%)
Minimum intensity	3 (16.7%)	2 (11.1%)	13 (72.2%)
Jitter	4 (22.2%)	9 (50%)	5 (27.8%)

	No dysphonia	Low-grade	Moderate	Severe
DSI	4 (22.2%)	7 (38.9%)	6 (33.3%)	1 (5.6%)

The Dysphonia Severity Index (DSI) is for perceptually normal voices equals + 5 and for severely dysphonic voices − 5. The average value of DSI in our patient collective was 2.81. This corresponds to a worse voice than normally. In detail, it was found “no dysphonia” only in four patients (22%). Most patients suffered from mild (7/39%), moderate (6/33%), or even severe (1/6%) hoarseness.

No patient had an inconspicuous voice in the auditory assessment (Table 5). In one patient (5.6%), even a severe hoarseness was present. The majority of the patients suffered from a slight impairment (14/77.8%). The other parameters assessed by auditory perception were most often affected by grade one. 72% suffered from a rough voice and 83% from a breathy voice. The voice was weak in 83% and with reduced tension in 66%.

**Table 5:** Auditory perceptual analysis (*n* = 18)

	0	1	2	3
G (grade)	0 (0%)	14 (77.8%)	3 (16.7%)	1 (5.6%)
R (rough)	2 (11.1%)	13 (72.2%)	3 (16.7%)	0 (0%)
A (asthenic)	3 (16.7%)	13 (72.2%)	2 (11.1%)	0 (0%)
B (breathy)	3 (16.7%)	11 (61.1%)	4 (22.2%)	0 (0%)
S (strained)	6 (33.3%)	10 (55.6%)	2 (11.1%)	

**Fig. 1:**
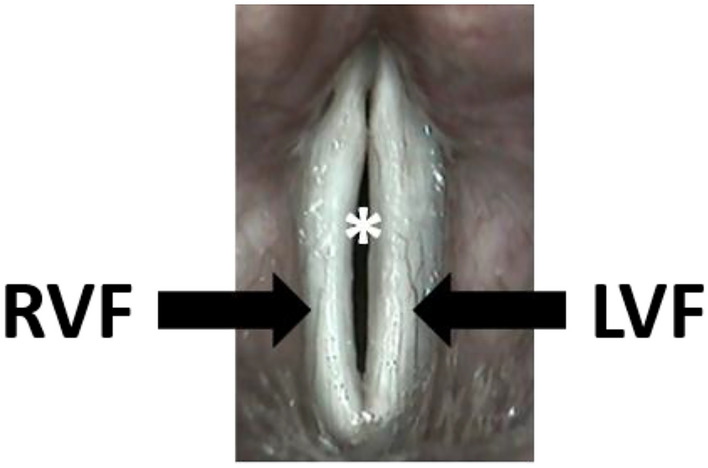
Videostroboscopy: the white vocal folds are in the phonation position. However, they are not completely closed (*), because the right vocal fold (RVF) is in the opening phase and the left vocal fold (LVF) is still in the closing phase. This is an expression of an abnormal symmetry that causes hoarseness

We detected few morphological abnormalities (laryngeal mucus, vocal folds with distinctive vessels and/or edema) in the laryngoscopic examination. In contrast, we often observed changes in the vibration characteristics of the vocal folds in videostroboscopy (Fig. 1) (Table 6). We noted an incomplete glottis closure (71%), a changed amplitude (65%), irregular oscillation sequences (41%), a limited mucosal wave (94%), and phase shifts in symmetry (88%). Due to a severe gag reflex, the videostoboscopy of one proband could unfortunately not be evaluated.

**Table 6:** Laryngeal findings in videolaryngoscopy and videostroboscopy (*n* = 17)

Videolaryngoscopy	Present	Absent
Laryngeal mucus	5 (29.4%)	12 (70.6%)
Vocal folds with distinctive vessels	8 (47.1%)	9 (52.9%)
Vocal folds with edema	5 (29.4%)	12 (70.6%)

Videostroboscopy	Normal	Altered
Glottis closure complete	5 (29.4%)	12 (70.6%)
Amplitude	6 (35.3%)	11 (64.7%)
Irregular oscillation sequences	10 (58.2%)	7 (13%)
Mucosal wave	1 (5.9%)	16 (94.1%)
Symmetry	2 (11.8%)	15 (88.2%)

## Discussion

In the present study, we examined patients with SjS and determined the swallowing-, and voice-related quality of life and additionally conducted a detailed voice examination.

The questionnaires and examinations used have been established for many years and include well-known standard values or grade classifications for normal populations. Like most of the other research groups, we also decided not to test a healthy control group [24, 25, 28, 29].

The Anderson Dysphagia Inventory (ADI) is validated in Germany for oral cancer. We have decided to use it based on its better reflection of swallowing disorders caused by dryness of the mouth and the pharynx, as they occur in head and neck tumors due to surgery and radiation, than the quality of life questionnaires that focuses on neurogenic swallowing disorders (e.g. SWAL-QOL) [29]. Other studies have also applied the ADI in SjS to assess the quality of life related to swallowing, which enables the comparison of results [10, 30].

In our study, the quality of life assessment shows that almost all patients (96.3%) are moderately to severely impaired by their swallowing problems. This correlated significantly with xerostomia; the higher the dryness, the more limited was the swallowing-related quality of life. A further indication of dryness in our patient collective was that the ESSPRI was elevated overall, but it was the category of dryness that was most severely affected.

As it is one of the cardinal symptoms of SjS, a very high percentage of patients suffers from xerostomia (91.7–100%) [31, 32]. Xerostomia interferes with the oral transport and the pharyngeal phase of swallowing. This may increase residues especially with solid and semi-solid foods in the mouth and the pharynx [33]. In SjS, it is mainly the swallowing efficiency and only in a smaller degree the swallowing safety, which is affected [34]. There are few penetrations or aspirations with lethal pneumonia as a sign of swallowing safety. Although there are no life-threatening complications of swallowing in SjS patients, the quality of life is considerably reduced by the swallowing problems. We, therefore, recommend that SjS patients with dry mouth and high xerostomia values receive further swallowing diagnosis, e.g. fiberoptic evaluation of swallowing (FEES) [11]. The exact results of the swallowing examination can be used to initiate an adequate logopaedic swallowing therapy based on the pathomechanisms of the disorder. The therapy can thus improve swallowing efficiency and improve the quality of life.

The anatomical parts of the mouth and the pharynx are not only used for swallowing but are elementary structures for the production of voice. The dryness of these structures therefore also affects the voice. We found that the ADI correlated significantly with the voice-related quality-of-life (VHI) as well as in other studies [35]. The limitations of voice-related quality of life were less severely than swallow-related quality of life. The voice is an important instrument of expression in our communication society [36]. Hoarseness occurs when the two vocal folds do not close and each vocal fold vibrates irregularly at its own frequency. This can be a consequence of visible organic changes in the vocal folds, seen in a routine ENT medical examination using a laryngoscopy. Also, irregular oscillations of the vocal fold mucosa cause hoarseness. These irregular oscillation sequences are examined by videostroboscopy. Voice production is a complex phenomenon that involves not only the larynx but the entire vocal tract. The vocal tract is defined by all airy spaces above the vocal folds, in particular the supraglottis, the pharynx, the oral cavity and the nose. Voice disorders result from dysfunctions of these structures [13]. It is not surprising that changes in the vibrational properties of these structures, such as those caused by SjS, lead to voice disorders. Different changes in the voice quality are found with SjS [14, 15].

Since many factors influence the voice, the examination of the voice follows a multidimensional approach recommended by the European Laryngological Society (ELS) [3].

Although the vast majority of patients had a slightly decreased max. phonation time (67%), the phonation quotient was normal or even better in more than half of the patients. Only one other study measured the aerodynamic parameters as well and was also not able to find any statistically significant changes [14].

In the voice range profile, most patients were able to achieve a normal pitch (56%) and dynamic range (72%). Our results for jitter, a measurement of irregularities in the frequency range, showed abnormal values in more than half of the patients. The Dysphonia Severity Index (DSI), which is calculated from various parameters, showed no dysphonia in only 22%. Most patients suffered from a mild (39%), moderate (33%) or even severe (6%) hoarseness. No other research groups carried out these studies in patients with SjS. We believe that the multidimensional DSI can provide valuable information about a voice disorder and at the same time determine a degree of severity.

In the videolaryngoscopic examination, we found hardly any morphological abnormalities (laryngeal mucus, vocal folds with distinctive vessels or swellings). In a further examination using videostroboscopy, changes in the vibration behavior of the vocal folds were detected, which cause hoarseness. Almost all parameters showed abnormalities: an incomplete glottis closure (71%), changed amplitude (65%), limited mucosal wave (94%), an abnormal symmetry of the oscillation phase (88%). Other studies, which have also investigated changes in the vibrational behavior of the vocal folds, come to similar findings [12, 15, 37, 38].

Although the parameters are affected differently in the few investigations of this topic, the results show that, on a general view, all of them could find no morphological changes in the laryngoscopy, but all of them found a disturbed swinging behavior of the vocal folds in the stroboscopy. A differentiated examination of the voice should therefore always include a stroboscopic examination.

One possible therapy for voice disorders is voice therapy. Although few patients with SjS are receiving voice therapy until now, the majority of those who did benefited significantly from it. [38]. In our study, the ADI, VHI and EORTC QLQ H&N 35 correlated statistically significantly with xerostomia. It is obvious that xerostomia can cause voice and swallowing disorders. At the moment there is no known therapy that can restore glandular function [16]. Therefore, the first therapeutic approach for dryness should be symptomatic relief using topical therapies, for example liposomal local therapy [39–41].

Our cohort of patients has a special distribution regarding positivity of antibodies and histological proof. Both the rates of 37% positivity for SS-A and 26% positivity for SS-B are comparatively low compared to positivity rates usually reported in the literature. Most of our patients are referred to our clinic in the case of negativity for SS-A and SS-B but still suspected Sjögren’s syndrome for further evaluation by minor salivary gland biopsies. This explains the low rate of antibody positivity in this cohort and the high rate of conducted biopsies. A matched control group would have been beneficial to identify changes caused by the SjS. The aim of this study was to get a first insight in voice and swallowing disorders in patients with SjS and for the identification of changes/abnormalities we used validated questionnaires and objective findings. Swallowing and voice changes may be influenced by the age of the patients [1],^2^.The median age of the included subject was 58 years and, therefore, no relevant age-related changes in swallowing or voice function was assumed.

In conclusion, the current study demonstrates the reduction of the swallowing- and voice-related quality of life and frequency of voice disorders in SjS. We suggest, that voice and swallowing difficulties should be evaluated on a regular basis in addition to xerostomia. The more detailed the diagnosis, the more specific a therapy can be. Patients with a reduced quality of life related to swallowing and/or voice disorders should be referred to a detailed laryngological examination by an ENT physician or phoniatrician. However, while this evaluation provides a first insight in voice and swallowing disorders in patients with Sjögren’s syndrome, the provided evidence is limited due to the scarceness of included patients.
